# Gut Leakage Markers in Response to Strenuous Exercise in Patients with Suspected Coronary Artery Disease

**DOI:** 10.3390/cells10092193

**Published:** 2021-08-25

**Authors:** Susanne Kristine Aune, Joanna Cwikiel, Arnljot Flaa, Harald Arnesen, Svein Solheim, Ayodeji Awoyemi, Marius Trøseid, Ingebjørg Seljeflot, Ragnhild Helseth

**Affiliations:** 1 Center for Clinical Heart Research, Department of Cardiology, Oslo University Hospital Ullevål, 0424 Oslo, Norway; jcwikiel@gmail.com (J.C.); UXHAAR@ous-hf.no (H.A.); SSOLHEIM@ous-hf.no (S.S.); sbawoy@ous-hf.no (A.A.); uxinlj@ous-hf.no (I.S.); ramhel@ous-hf.no (R.H.); 2 Faculty of Medicine, University of Oslo, 0316 Oslo, Norway; marius.troseid@medisin.uio.no; 3 Department of Cardiology, Oslo University Hospital Ullevål, 0424 Oslo, Norway; UXFARN@ous-hf.no; 4 Section of Clinical Immunology and Infectious Diseases, Oslo University Hospital Rikshospitalet, 0424 Oslo, Norway

**Keywords:** gut leakage, microbiota, cardiovascular disease, inflammation, acute exercise, strenuous exercise

## Abstract

Elevated levels of gut leakage markers have been shown after strenuous exercise in healthy individuals. Any association between a temporary increase in these markers and the presence of coronary artery disease (CAD) is unknown. We therefore aimed to explore circulating gut leakage markers in response to a bout of strenuous exercise in patients with symptoms of CAD. Patients referred to exercise stress testing due to symptoms of CAD were included (*n* = 287). A maximal exercise ECG stress test was performed and venous blood samples were drawn at rest and within five minutes after, for analysis of soluble cluster of differentiation 14 (sCD14), lipopolysaccharide-binding protein (LBP), intestinal fatty-acid binding protein (I-FABP), lipopolysaccharide (LPS) and gene expression of toll-like receptor 4 (TLR4) in circulating leukocytes. Patients then underwent coronary angiography. LPS, LBP and sCD14 increased significantly after strenuous exercise in patients with symptoms of CAD, suggesting that even short bouts of vigorous exercise are associated with gut leakage. The gene expression of TLR4 decreased significantly after exercise, possibly as a negative feedback to the increase in LPS. There were no differences in exercise-induced changes between the groups of CAD, suggesting gut leakage to be independent of the presence of CAD.

## 1. Introduction

Regular physical activity is associated with reduced risk for cardiovascular disease (CVD) [1,2]. Regular endurance exercise has been shown to reduce biomarkers associated with chronic, vascular inflammation and the development of atherosclerosis such as tumor necrosis factor alpha (TNF-α), interleukin 6 (IL-6) and C-reactive protein (CRP) [3]. Additionally, regular exercise has been shown to beneficially alter the composition and quality of the gut microbiota, preventing gut dysbiosis and reducing circulating gut leakage markers [4,5]. Gut microbiota alterations have in several studies been linked to cardiometabolic risk factors and presence of CVD [6].

Acute strenuous exercise, as opposed to regular exercise, is associated with elevated risk for coronary events, especially in individuals with pre-existing CAD [1,7]. Established underlying mechanisms are suggested to include increased arterial wall sheer stress, coronary artery spasms, increased flexing of the coronary arteries causing plaque disruption, as well as systemic changes such as increased thrombogenicity and platelet activation [8]. 

After strenuous exercise, a transient increase in gut leakage markers, including lipopolysaccharide (LPS), has been observed [9,10]. LPS is a potent activator of inflammatory pathways through its interactions with LPS-binding protein (LBP), soluble cluster of differentiation 14 (sCD14) and toll-like receptor 4 (TLR4) on monocytes and macrophages [11]. Strenuous exercise may lead to gut leakage by affecting the intestinal wall integrity through alterations of the epithelial cells and tight junctions (TJs) [12], or through reduced splanchnic blood flow causing intestinal ischemia, epithelial cell damage and leakage of the intracellular intestinal fatty-acid binding protein (I-FABP) into the circulation [13]. However, the majority of research on exercise and gastrointestinal integrity and leakage is performed in healthy, endurance-trained populations [14]. The knowledge on gut leakage after strenuous exercise in patients with symptoms of CAD is scarce or lacking. 

In this study, we aimed to explore gut leakage markers in response to acute strenuous exercise in patients with symptoms of CAD. We hypothesized that gut leakage markers would increase in all subjects after the exercise, and that the increase would be more pronounced in patients with verified CAD. 

## 2. Materials and Methods

### 2.1. Study Population

Patients referred to an outpatient exercise stress test (EST) due to symptoms suggestive of CAD (*n* = 327) were included in the CADENCE study (clinicaltrials.gov NCT01495091), at the Department of Cardiology, Oslo University Hospital Ullevaal, Oslo Norway between December 2011 and October 2017 [15], of which this is a sub study.

Details of the study design have previously been published [15]. In brief, patients ≥18 years of age with symptoms suggestive of CAD and an intermediate or high pre-test prognostic risk score (Morise score ≥ 9 points [16]) were included. Exclusion criteria were acute coronary syndrome, clinical heart failure, on-going arrhythmia or implanted pacemaker, moderate to severe valvular heart disease, prior coronary artery bypass graft (CABG) surgery, renal insufficiency (S-creatinine > 150 μmol/L), or inability to perform exercise testing or coronary angiography.

A physical examination including blood pressure and weight, and a resting 12-lead ECG was performed in all patients prior to the exercise stress testing. Hypertension and hypercholesterolemia were defined according to established diagnosis or the use of relevant medications. 

All participants gave written informed consent to participate. The study has been conducted in accordance with the Declaration of Helsinki, and the Regional Ethics Committee of the South Eastern Norway Regional Health Authority approved the protocol.

### 2.2. Exercise Stress Test

An EST was performed on an electrical bicycle ergometer (Ergoline GmbH, Bitz, Germany and Schiller CS-200 Excellence, Baar, Switzerland), monitored by a physician and nursing staff. Continuous 12-lead computerized ECG monitoring was used during the test. The EST has previously been described in detail [15]. In brief, patients were instructed to maintain a pedalling rate of around 65 rpm, while the workload was gradually increased by 10 W every min. The participants were asked about their perceived exertion every three min, using the Borg scale of rated perceived exertion (RPE) [17]. If no clinical signs of ischemia developed before reaching high intensity level, the patients were exercised to exhaustion, and the test was stopped after 5 min recovery. Development of ECG changes such as ST-segment elevation or ST-segment depression in leads without Q waves, arrhythmias increasing through exercise, chest pain, insufficient chronotropic response to exercise, and insufficient or exaggerative hypertensive response (systolic blood pressure ≥ 250 mmHg or diastolic blood pressure ≥ 115 mmHg) were reasons for active termination of the test [18]. 

### 2.3. Laboratory Methods

Blood samples were drawn at rest prior to exercise testing and within 5 min of terminating workload, while the patients were still seated on the bicycle ergometer. EDTA plasma were kept on ice until separated within 30 min by centrifugation at 2500× *g* at 4 °C for 20 min and serum was prepared by centrifugation within 1 h at 2000× *g* for 10 min at room temperature. PAXgene tubes (PreAnalytix GmbH, Hombrechtikon, Switzerland) were collected from 101 randomly selected patients for RNA extraction from circulating leukocytes. All samples were kept frozen at −80 °C until analyses. 

I-FABP was determined in serum by the commercially available enzyme-linked immunosorbent assay (ELISA) (Hycult Biotech, Uden, The Netherlands). sCD14 and LBP were measured in EDTA plasma by ELISAs (R & D Systems Europe, Abingdon, Oxon, UK and Hycult Biotech, respectively). LPS was measured in EDTA plasma using the Kinetic Chromogenic Limulus Amebocyte Lysate (LAL) Assay, Lonza BioScience, Basel, Switzerland in the same subset of patients (*n* = 101) available for gene expression analyses of TLR4. The inter-assay coefficients of variation (CV) were 7.9%, 8.1%, 11.5% and 11% respectively. 

For gene expression of TLR4, total RNA was isolated from PAXgene tubes using PAXgene^®^ Blood RNA Kit (PreAnalytix, Qiagen, GmBH, Hombrechtikon, Switzerland), including an extra cleaning step (RNeasy^®^ MinElute^®^ Cleanup Kit, Qiagen). The quality and quantity (ng/µL) of RNA was examined by the NanoDropTM 1000 Spectrophotometer (Saveen Werner, Thermo Scientific, Wilmington, DE, USA). Copy DNA (cDNA) was created by mixing equal amounts of RNA (5 ng /µL) and qScriptTM cDNA superMix (Quanta Biosciences, Gaithersburg, MD, USA). The gene expression was analysed by real time PCR performed on a ViiATM7 instrument (Applied Biosystems by Life Technologies, Foster City, CA, USA), using TaqMan^®^ Universal PCR Master Mix (P/N 4324018) with a commercially available TaqMan^®^ assay: TLR4 (Hs00152939_m1) (Applied Biosystems by Life Technologies). mRNA levels were determined with the ∆∆CT method, using β2-microglobulin (HS99999907_m1) (Applied Biosystems by Thermo Fisher Scientific, Life Technologies Corporation, Pleasanton, CA, USA) as an endogenous control and related to a reference sample, giving relative quantification (RQ) [19]. 

### 2.4. Coronary Angiography

All patients underwent coronary angiography, mostly by radial artery access. An interventional cardiologist performed and described all coronary angiograms and a single investigator thereafter went through all procedure descriptions. For the purpose of this investigation, patients were divided into three groups: (I) *Significant* CAD was defined as (i) ≥75% stenosis in any coronary segment, (ii) any stenosis the angiographer considered clinically relevant or decided should be treated with PCI or CABG surgery and (iii) previous PCI; (II) *Non-significant* CAD included patients with the range of atherosclerotic changes from minimal wall changes to <75% stenosis, and (III) *No* CAD was defined as no wall changes or stenotic segments. If the investigator was in doubt, a senior investigator was consulted.

### 2.5. Statistical Analysis

Data are given as mean (±SD), median (25th and 75th percentiles) or proportions as appropriate. Differences between groups of three were analysed by the Kruskal-Wallis test, while the Mann-Whitney U-test was performed for differences between any two groups. Correlation analyses were performed by Spearman’s Rho. Changes in biomarkers from before to after the EST, delta values, are given as absolute and relative changes, and analysed by the Wilcoxon matched pairs Signed Rank test. Statistical calculations were performed using Stata SE version 15 (StataCorp LLC, College Station, TX, USA). *p*-values < 0.05 were considered statistically significant.

## 3. Results

### 3.1. Patient Characteristics

Baseline characteristics for the total cohort and according to groups of CAD are presented in Table 1. Of 297 patients completing the EST and coronary angiography, 10 patients were excluded due to lack of blood samples. Thus, 287 patients (*n* = 287) make up the total study population. 

As outlined in Table 1, 69 patients (24%) had no CAD, 88 patients (30.7%) had non-significant CAD and 130 patients (45.3%) had significant CAD. There were no statistically significant differences in age or body mass index (BMI) between groups of CAD, although patients with significant CAD tended to be older than patients with no CAD (*p* = 0.037). There were more women in groups with no or non-significant CAD, the difference was significant between all groups of CAD (*p* < 0.001). Risk factors such as smoking, diabetes and hypertension were equally distributed in groups of CAD, although the presence of hypertension tended to be more common in patients with significant CAD compared to patients with no CAD. The presence of hypercholesterolemia was statistically different between all groups of CAD, and was more prominent in the groups with non-significant and significant CAD. There was significantly higher usage of platelet inhibitors, betablockers and calcium blockers along more pronounced CAD, and the difference was significant between all groups of CAD (Table 1). There was a higher usage of statins along more pronounced CAD, significant between all groups of CAD (*p* < 0.001). Total cholesterol and LDL levels were statistically lower in groups with CAD compared to no CAD, both when comparing no CAD to non-significant and significant CAD together, and to significant CAD alone (Table 1).

### 3.2. Exercise Performance

As outlined in Table 2, the median exercise duration was 09:31 min in the total population, and did not differ significantly between the groups. Medians of metabolic equivalent (METs) and maximal RPE on the Borg scale were both significantly lower in patients with CAD. METs were significantly lower only between no CAD and significant CAD (Table 1). 172 patients (60%) reached Borg Scale ≥ 17, and there was no significant difference between the groups (*p* = 0.15) (data not shown). 

### 3.3. Gut Leakage Markers at Baseline

As shown in Table 3, there was no significant difference in baseline levels of the gut leakage markers between the three groups of CAD. As to anthropometric measures, LBP correlated significantly to BMI, waist circumference and weight (rho = 0.26, 0.38 and 0.38, *p* < 0.001 for all). In patients with BMI ≥ 25 kg/m^2^, LBP was significantly higher (*p* = 0.019) than in those considered normal weight (BMI < 25 kg/m^2^). There were no significant differences in gut leakage markers according to presence of diabetes. 

sCD14 and LBP correlated inversely to METs (rho = −0.15 and −0.31, *p* = 0.015 and *p* < 0.001, respectively) and exercise duration (rho = −0.25 and −0.15, *p* < 0.001 and *p* = 0.014, respectively) in all patients. LPS, I-FABP and TLR4 did not correlate to exercise capacity measured by METs and exercise duration (*p* > 0.05, all). There was no significant correlation to maximal RPE for any of the investigated markers (*p* > 0.05, all).

### 3.4. Gut Leakage Markers and Strenuous Exercise

**Figure 1:**
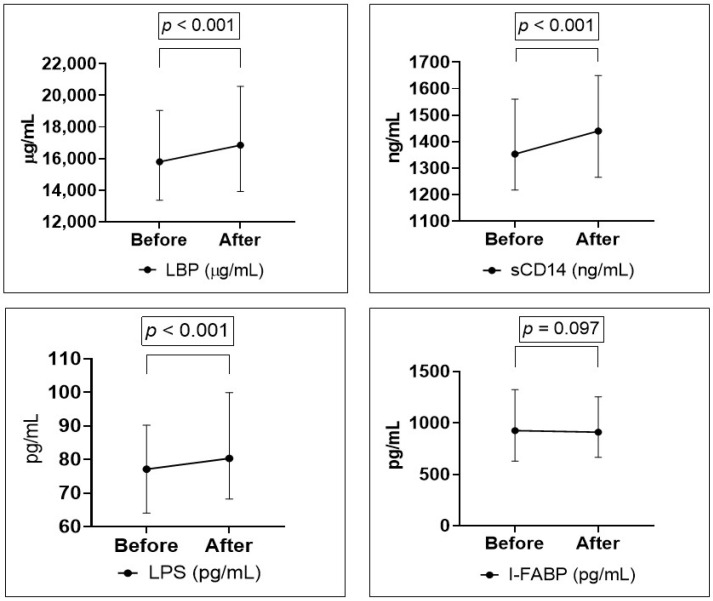
Change in gut leakage markers LBP, sCD14 and I-FABP from before to after exercise in all patients (*n* = 287), and LPS in a subset of patients (*n* = 101).

Values of gut leakage markers before and after strenuous exercise in the total population are presented in Figure 1. LPS, LBP and sCD14 increased significantly in all patients. I-FABP did not change significantly from before to after the EST. 

**Figure 2:**
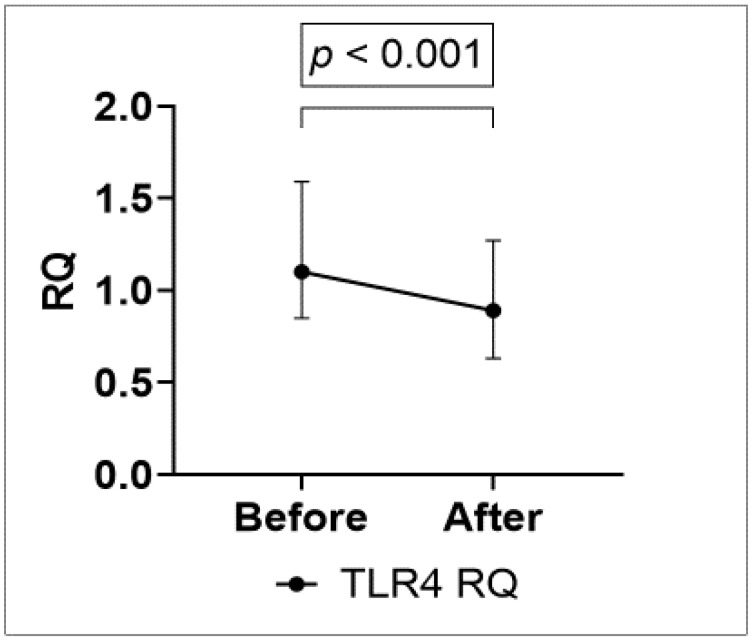
Change in relative gene expression of TLR4 from before to after exercise in a subset of patients (*n* = 101).

As LBP and sCD14 are typically released from leucocytes upon LPS stimulation of the TLR4 complex, we next investigated whether TLR4 expression on leucocytes was altered. As depicted in Figure 2, gene expression of TLR4 in leucocytes decreased with approximately 22% (*p* < 0.001) after strenuous exercise (Figure 2). There was no significant difference in change in gut leakage markers according to the presence of diabetes or overweight (BMI ≥ 25 kg/m^2^) (data not shown).

As outlined in Table 4, there was no significant difference in change in gut leakage markers between any groups of CAD, nor was there any significant difference in change in gut leakage markers between no CAD and non-significant and significant CAD together (*p* > 0.05, all) or no CAD and significant CAD alone (*p* > 0.05, all). There were no significant differences in relative changes either (Table 4). 

## 4. Discussion

The main findings of the present study were that most of the investigated gut leakage markers increased immediately after strenuous exercise in patients with symptoms of CAD, indicating that even short bouts of vigorous activity are associated with gut leakage. Gut leakage markers were not differently expressed in patients eventually shown to have angiographically verified CAD. 

### 4.1. Effect of Exercise on Gut Leakage Markers

LPS, LBP and sCD14 all increased significantly as a result of a short bout of strenuous exercise, suggesting an LPS-driven activation of the LPS-LBP-sCD14 inflammatory pathway. Indeed, we have previously reported on increased inflammatory markers, reflecting generalized inflammation, in a subset of the investigated population [20]. Our findings are in line with previous studies, although occurring after significantly shorter exercise duration (≈10 min) in our population compared to the threshold at which gastrointestinal perturbations has been suggested to manifest (>2 h) [14]. However, in the aforementioned review the vast majority of participants were healthy, trained individuals, which might explain the difference to our observations. To our knowledge, this is the first study reporting such a relationship in patients with symptoms of CAD. 

As opposed to LPS, LBP and sCD14, gene expression of TLR4 was reduced in all patients after exercise. One explanation might be a rapid increase in translation of cytosolic TLR4 mRNA to TLR4 protein, as a response to increased concentration of LPS. However, it has been shown that the cell surface expression of TLR4 in monocytes is reduced immediately after exercise [21], while others also have reported on a reduction in TLR4 gene expression after maximal exercise, in line with our result [22]. One might speculate that increased LPS concentrations induce a negative feedback on the TLR4 gene expression. Previous research has, however, demonstrated elevated protein levels as well as mRNA levels of TLR4 after stimulation with LPS in vitro [23]. It is therefore not necessarily LPS that induces the reduction in TLR4 gene expression in our patients. It has been hypothesised that exercise-induced IL-6, which have both pro- and anti-inflammatory properties, down regulate the gene expression of TLR4 [21]. An increase in IL-6 was indeed found in our patients, as previously reported [20]. It could also be a result of the anti-inflammatory effect of cortisol [24].

There was no change in I-FABP from before to after the EST. As I-FABP is an intracellular protein, it increases in the circulation in the case of intestinal cell damage [25]. Gut leakage observed in this study after a short bout of exercise is therefore probably not due to intestinal ischaemia or enterocyte damage, but rather occurs through altered TJs leading to paracellular leakage or through increased transcellular uptake. As diabetic and overweight patients have altered uptake of fatty acids and chylomicrons [26], we hypothesized that if increased gut leakage was due to altered uptake of LPS, gut leakage markers would increase more in these patients. We found no such difference in either of the two groups. Exercise training, on the other hand, may alter the TJs by phosphorylation [12], and it is therefore tempting to speculate whether that is the mode of action in this case. Also, important inflammatory cytokines increase the TJ permeability [27], which could support the theory of a transiently altered paracellular permeability in our patients. 

Our results allow us to speculate that the transitory inflammatory activation associated with acute exercise might in part be due to gut leakage, in line with other studies that have reported an increase in both LPS and pro-inflammatory cytokines after strenuous exercise [28]. It is, however, debated whether the increase in inflammatory markers associated with acute exercise actually reflects a net pro-inflammatory environment [29]. It has been suggested that single bouts of strenuous exercise in summary contribute to an anti-inflammatory milieu, particularly through increase in IL-6 as well as IL-6 receptor from contracting skeletal muscle [30]. The reduction in TLR4 gene expression in our patients may therefore advocate that although accompanied by an increase in gut leakage markers, a single bout of strenuous exercise leads to an increased, but partly counterbalanced, inflammatory advertence. 

The long-term effects of strenuous exercise on gut associated increase in inflammatory markers remains unknown. It has been shown that healthy individuals exercising on a regular basis had higher levels of anti-LPS [9], indicating a form of self-immunization against regular LPS exposure. Additionally, we found higher baseline levels of sCD14 and LBP in patients whit poorer physical capacity in the EST, in line with our previous findings in a population with both CAD and diabetes type 2 [31]. As regular exercise decreases the risk for exercise-related coronary events [32], one might therefore speculate that the reduction in risk, in part, lies in the immune system’s ability to deal with exercise-induced gut leakage. However, as we in this study investigate only a single bout of strenuous exercise, it is not suitable for studying any long-term effects of regular exercise.

### 4.2. Difference between Groups of CAD

There was no difference in changes from before to after exercise between the three groups of CAD. As gut dysbiosis and an altered gut microbial environment is a common feature of CVD [33], we had hypothesised patients with any CAD to have a more pronounced gut leakage after strenuous exercise than patients with no CAD. However, such a relationship could not be demonstrated in our population. The population with any CAD had significantly higher usage of medication known to interfere with the gut microbial composition such as statins and acetylsalicylic acid, potentially masking any difference. No significant difference in change was found between no CAD and significant CAD alone either. 

Our results indicate, however, that gut leakage occurs in all subjects after strenuous exercise, irrespective of the presence of CAD, and that the difference in its consequence possibly lies in the body’s ability to handle the stress of transiently increased LPS and associated inflammatory activation.

## 5. Limitations

This study was initially designed to investigate cardiac biomarkers during an EST, such as troponin T, as additional information for the diagnosis of CAD, and thus not powered for the present investigation [15]. The study should be regarded as explorative and hypothesis generating. We decided not to adjust for changes in haematocrit, as we believe that the unadjusted values reflect the actual and clinically relevant concentration of gut leakage markers in our patients. Factors known to interfere with the gut microbiota, e.g., diet and use of drugs such as antibiotics, proton pump inhibitors and immunomodulatory medication, have not been controlled for [26].

## 6. Conclusions

In our patients with symptoms of CAD, a short bout of strenuous exercise led to increased levels of the gut leakage markers LPS, LBP and sCD14, and reduced gene expression of TLR4, independent of the presence of CAD. The reduction in TLR4 gene expression might be a counterbalanced response to the transient increase in inflammatory activation. The lack of increase in I-FABP may indicate that gut leakage during such short bouts of exercise is not due to intestinal ischemia, but rather reflect either para- or transcellular leakage.

## Figures and Tables

**Table 1:** Baseline characteristics of the total population and according to presence of CAD.

	All (*n* = 287)	*No* CAD (*n* = 69)	*Non-Significant* CAD (*n* = 88)	*Significant* CAD (*n* = 130)	*p*-Value ^1^	*p*-Value ^2^	*p*-Value ^3^
Age (years) *	62.4 (40.1–87.2)	60.8 (43.8–80.6)	61.7 (40.1–79.6)	63.7 (40.9–87.2)	0.079	0.085	**0.037**
**Sex; female, *n* (%)**	102 (35.5)	44 (63.8)	32 (36.4)	26 (20.0)	**<0.001**	**<0.001**	**<0.001**
**BMI (kg/m^2^)**	26.6 (24.7, 29.6)	26.8 (24.3, 30.4)	26.3 (24.7, 29.0)	26.6 (25.0, 30.0)	0.271	0.954	0.524
**Diabetes, *n* (%)**	56 (19.5)	15 (21.7)	12 (13.6)	29 (22.3)	0.248	0.593	0.927
**Hypertension, *n* (%)**	169 (58.9)	34 (49.3)	50 (56.8)	85 (65.4)	0.081	0.063	**0.041**
**Resting heart rate (bpm)**	68 (61, 77)	70 (62, 75)	70 (62, 80)	67 (59, 75)	0.076	0.630	0.189
**Hypercholesterolemia, *n* (%)**	231 (80.5)	42 (60.9)	71 (80.7)	118 (90.8)	**<0.001**	**<0.001**	**<0.001**
**Smoker, *n* (%) ^4^**	52 (18.1)	39 (56.5)	54 (61.4)	83 (63.9)	0.602	0.348	0.391
**HbA1c %**	5.7 (5.4, 6.2)	5.7 (5.4, 6.2)	5.6 (5.4, 5.9)	5.7 (5.4, 6.3)	0.232	0.720	0.753
**Creatinine (μmol/L)**	79 (69, 89)	73.5 (66, 80.5)	78 (68, 88)	82 (73, 93)	**<0.001**	**<0.001**	**<0.001**
**Total cholesterol (mmol/L)**	4.6 (3.9, 5.6)	5.3 (4.5, 6.2)	4.7 (3.9, 5.8)	4.3 (3.7, 5)	**<0.001**	**<0.001**	**<0.001**
**LDL (mmol/L)**	2.6 (2.1, 3.5)	3.0 (2.4, 4.0)	2.6 (2.0, 3.6)	2.4 (2.0, 3.0)	**0.001**	**0.002**	**<0.001**
**Medication, *n* (%)**							
**Platelet inhibitor ^5^**	209 (72.8)	29 (42.0)	63 (71.6)	117 (90.0)	**<0.001**	**<0.001**	**<0.001**
**ACEI/ARB**	103 (35.9)	17 (24.6)	35 (39.8)	51 (39.2)	0.083	**0.026**	0.054
**Betablocker**	119 (41.5)	14 (20.3)	34 (38.6)	71 (54.6)	**<0.001**	**<0.001**	**<0.001**
**Calcium blocker**	47 (16.3)	5 (7.3)	14 (15.9)	28 (21.5)	**0.035**	**0.019**	**0.013**
**Statins**	203 (70.7)	34 (49.3)	60 (68.2)	109 (83.9)	**<0.001**	**<0.001**	**<0.001**

Variables are given as medians (25th, 75th percentile) unless stated otherwise. * Mean (min, max). ^1^ refers to the difference between the groups of CAD. ^2^ Refers to differences between no CAD and any CAD (non-significant CAD and significant CAD together). ^3^ refers to differences between no CAD and significant CAD. ^4^ Includes smokers and previous smokers. ^5^ Refer to the use of acetylsalisylic acid, dipyramidol or clopidogrel, or combinations of these. BMI = body mass index, bpm = beats per minute, LDL = low-density lipoprotein, ACEI = angiotensin-converting-enzyme inhibitor, ARB = angiotensin II receptor blocker.

**Table 2:** Exercise performance in all patients and according to coronary artery disease.

	All (*n* = 287)	*No* CAD (*n* = 68)	*Non-Significant* CAD (*n* = 88)	*Significant* CAD (*n* = 130)	*p*-Value ^1^	*p*-Value ^2^	*p*-Value ^3^
**Exercise duration (min:sec)**	09:31 min (07:20, 12:04)	10:00 min (8:04, 13:24)	09:54 min (07:24, 11:50)	09:16 min (06:43, 12:03)	0.154	0.099	0.070
**METs**	6.7 (5.7, 8.0)	7.0 (6.1, 8.2)	7.0 (5.8, 8.0)	6.3 (5.6, 8.0)	**0.036**	0.096	**0.023**
**Maximal RPE, Borg scale**	17 (15, 18)	17 (16, 19)	17 (15, 18)	17 (15, 17)	**0.010**	**0.037**	**0.006**

^1^ difference between groups of CAD. ^2^ Refers to differences between no CAD and any CAD (non-significant CAD and significant CAD together). ^3^ refers to differences between no CAD and significant CAD. METs = metabolic equivalents. RPE = rated perceived exertion. All variables are presented as medians (25th, 75th percentiles).

**Table 3:** Gut leakage markers at baseline in all patients and according to coronary artery disease.

	All (*n* = 287)	*No* CAD (*n* = 69)	*Non-Significant* CAD (*n* = 88)	*Significant* CAD (*n* = 130)	*p*-Value ^1^	*p*-Value ^2^	*p*-Value ^3^
**sCD14 (ng/mL)**	1353 (1217, 1560)	1419 (1224, 1600)	1402 (1220, 1607)	1329 (1194, 1495)	0.097	0.312	0.107
**LBP (ng/mL)**	15,805 (13,378, 19,045)	15,033 (12,276, 18,593)	15,562 (12,723, 19,032)	16,293 (13,877, 19,233)	0.415	0.318	0.232
**I-FABP (pg/mL)**	927 (631, 1324)	943 (677, 1287)	894 (600, 1341)	925 (629, 1292)	0.811	0.832	0.981
	All (*n* = 101)	*No* CAD (*n* = 29)	*Non-Significant* CAD (*n* = 27)	*Significant* CAD (*n* = 45)			
**LPS (pg/mL)**	77.1 (64.0, 90.2)	74.3 (60.9, 89.2)	78.5 (66.3, 94.0)	78.3 (67.9, 91.2)	0.661	0.372	0.441
**TLR4 receptor (RQ)**	1.1 (0.9, 1.6)	1.3 (0.9, 1.7)	1.1 (0.8, 1.6)	1.1 (0.9, 1.4)	0.403	0.180	0.199

^1^ refers to difference between groups of CAD. ^2^ Refers to differences between no CAD and any CAD (non-significant CAD and significant CAD together). ^3^ refers to differences between no CAD and significant CAD. All variables are presented as medians (25th, 75th percentiles).

**Table 4:** Changes (delta values) in gut leakage markers according to coronary artery disease.

	No CAD (*n* = 69)	Non-Significant CAD (*n* = 88)	Significant CAD (*n* = 130)	Δ*p*-Value ^1^	Rel Δ*p*-Value ^2^
Δ **sCD14 (ng/mL)**	98 (−2, 192)	74 (−35, 174)	82 (−7.5, 180)	*p* = 0.915	*p* = 0.860
Δ **LBP (ng/mL)**	954 (471, 1783)	1188 (419, 1997)	995 (446, 1966)	*p* = 0.645	*p* = 0.793
Δ **I-FABP (pg/mL)**	−40 (−188, 146)	−26 (−187, 114)	−29 (−166, 121)	*p* = 0.914	*p* = 0.924
	(*n* = 29)	(*n* = 27)	(*n* = 45)		
Δ **LPS (pg/mL)**	4.5 (1.4, 17.6)	4.1 (−2.84, 13.3)	7.8 (−3.8, 14.3)	*p* = 0.552	*p* = 0.464
Δ **TLR4 RQ**	−0.3 (−0.70, −0.07)	−0.1 (−0.48, 0.04)	−0.20 (−0.40, 0.02)	*p* = 0.158	*p* = 0.384

^1^ refers to the difference in change between all three groups. ^2^ refers to the difference in relative changes between all three groups. All variables are presented as medians (25th, 75th percentiles).

## Data Availability

The datasets used and analysed during the current study are available from the corresponding author on reasonable request.
